# Variation of High and Low Nucleic Acid-Content Bacteria in Tibetan Ice Cores and Their Relationship to Black Carbon

**DOI:** 10.3389/fmicb.2022.844432

**Published:** 2022-02-14

**Authors:** Guannan Mao, Mukan Ji, Baiqing Xu, Yongqin Liu, Nianzhi Jiao

**Affiliations:** ^1^Key Laboratory of Tibetan Environment Changes and Land Surface Processes, Institute of Tibetan Plateau Research, Chinese Academy of Sciences, Beijing, China; ^2^Center for the Pan-Third Pole Environment, Lanzhou University, Lanzhou, China; ^3^CAS Center for Excellence in Tibetan Plateau Earth Sciences, Chinese Academy of Sciences, Beijing, China; ^4^College of Resources and Environment, University of Chinese Academy of Sciences, Beijing, China; ^5^State Key Laboratory of Marine Environmental Science, Xiamen University, Xiamen, China

**Keywords:** black carbon, bacteria functional groups, early-warning indicators, Tibetan Plateau, temporal variation, ice cores

## Abstract

Nutrient enrichment caused by black carbon (BC) is a major ecological crisis in glacial ecosystems. The microbiological effects of BC were assessed in this study by using fluorescent fingerprinting assay based on flow cytometry (FCM) of bacterial communities with low (LNA) and high (HNA) nucleic acid-content bacteria. Here, we investigated a high-resolution temporal variation of bacterial abundance and LNA/HNA ratio in Tibetan ice cores. Our results revealed that bacterial abundance was proportional to the atmospheric BC on the glaciers. The shift of LNA functional groups to HNA functional groups in glaciers suggested BC emissions increased the proportion of highly active cells. In addition, distinct number of LNA and HNA functional groups was identified between the monsoon and non-monsoon seasons. Westerly winds with high amounts of BC accounted for high ratio of HNA functional groups during the non-monsoon season. In comparison, high moisture during the monsoon season decreased atmospheric BC loading, which increases the ratio of LNA functional groups. Correlations between BC and functional groups were very strong, showing that two functional groups may serve as early-warning indicators of microbiological effects of BC at low trophic level. Our approach provides a potential early-warning framework to study the influences of atmospheric BC on the glaciological community.

## Introduction

Probing the bioavailability of black carbon (BC) released into the atmosphere is pivotal to understanding their impacts on the climate and environment. BC could impact the local and regional environment by absorbing solar radiation (Reid et al., 2005) and deteriorating trophic status (Odhiambo and Routh, 2016) when they deposited onto the surface of glaciers. BC is considered highly chemically recalcitrant; however, some studies exhibited that the microbes in oligotrophic environments could use BC as a nutritional and carbon source (Cheng and Foght, 2007; Stubbins et al., 2012). Bacteria not only are living in extremely cold and oligotrophic environments (Liu et al., 2016b), but also participate in the regional environmental variability, such as nutrient migration and transformation of organic carbon (Falkowski et al., 2008; Madsen, 2011). Despite of the crucial roles of bacteria in geochemical processes, there are still limited data on specific bacteria endpoints responses to long-term environmental and climatic changes. Glacial ice cores could record soluble chemical substance and bacteria in chronologically deposited archives (Legrand and Mayewski, 1997; Xiang et al., 2009), which present historical data on a range of climate changes and anthropogenic activity to occur (Liu et al., 2016b; Santibanez et al., 2016).

The ecosystem of Tibetan Plateau is sensitive to global climate change (Xu et al., 2009a; Liu et al., 2016b). The Tibetan Plateau lies in the immediate vicinity of two developing countries, China and India, and thus is subjected to influence of anthropogenic activities (Xu et al., 2009b; Yao et al., 2012; Li et al., 2016). Tibetan ice cores provide a medium to understand the long-term microbial responses relative to climate changes and anthropogenic activities. The relationship between bacterial abundance and the atmospheric circulation was observed in samples of Tibetan ice cores (Zhang et al., 2007). Specifically, dust carried by the westerly winds leaded to a higher bacterial abundance (Yao et al., 2008; Chen et al., 2016), while wet scavenging of Indian monsoon decreased bacterial abundance (Zhang et al., 2007). In addition to atmospheric circulation, the anthropogenic activities also increased bacterial abundance. Previous studies showed that increasing of bacterial abundance was associated with the deterioration of trophic status, such as increasing industrial production activities, desertification of grasslands, and deposition of BC from southern Asia (Miteva et al., 2009; Hara and Zhang, 2012; Liu et al., 2016b). Thus, bacteria in the ice cores could be a sensitive biomarker of climate and environmental changes.

The flow cytometry (FCM) combined with fluorescent staining technique has been widely used to quantify and visualize bacteria in environmental samples (Liu et al., 2016a; Sharuddin et al., 2018). Bacteria can be broadly divided into LNA and HNA functional groups (i.e., FCM’s fingerprints; Besmer et al., 2017), based on the observed correlation between fluorescence intensity and cellular nucleic acid content (Gasol et al., 1999; Lebaron et al., 2002; Bouvier et al., 2007; Proctor et al., 2018). Moreover, the composition and proportion of HNA and LNA functional groups could vary, depending on their adaptation to environmental conditions, that is, the HNA functional groups were sensitive to changes in nutrient and carbon availability in the environment (Kaartokallio et al., 2013; Santos et al., 2019), whereas the LNA functional groups were commonly linked to oligotrophic ecosystems (Mary et al., 2006; Wang et al., 2009). Thereby, the shift between HNA and LNA functional groups may be a potential biological indicator for environmental and climate changes. Unfortunately, there is still a lack of information on the distribution and shift of LNA and HNA functional groups in the Tibetan Plateau.

The objective of this study was to elucidate that the influences of regional anthropogenic BC on LNA and HNA functional groups assessed using FCM technology. Atmospheric BC has been shown an impact on regional carbon budget (Wang et al., 2019), therefore, we hypothesize that increasing deposition of BC could transform ecological status and microbiol structure. To achieve this, two glacier ice cores from Tibetan Plateau were investigated for temporal variation of LNA and HNA functional groups during the past half-century. In particular, we combined microbiological studies with BC analysis of the glacier ice cores to infer the relationship between the LNA-to-HNA ratio and anthropogenic activity.

## Materials and Methods

### Sampling Sites

Two ice cores were drilled at accumulation zones of two glaciers on the south of Tibetan Plateau (Supplementary Figure 1). The Zuoqiupu Glacier ice core [ZQP, 96.92°E,29.21°N, 5600 m above sea level (m.a.s.l.)] was retrieved from Mt. Gangrigabusupply. The Noijinkangsang Glacier ice core (NJKS; 90.20°E, 29.04°N, 5950 m.a.s.l.) was taken from Mt. Noijin Kangsang. Data from meteorological stations located Mt. Gangrigabusupply and Mt. Noijin Kangsang showed that average annual air precipitation was 392 mm (from 1960 to 2006) and 797 mm (from 1960 to 2006), and annual air temperature was 2.8°C (from 1960 to 2006) and 12.1°C (from 1960 to 2006). NJKS and ZQP are influenced by distinct prevailing weather patterns. NJKS is strongly influenced by the monsoon in summer and by the westerly jet stream in winter (Tian et al., 2001). ZQP is heavily marine influenced, with oceanic moisture directly transported from the Bay of Bengal along the Brahmaputra River valley (Tian et al., 2001). In addition, the two glaciers should receive BC both from the south *via* the Indian monsoon during summer and from the west *via* westerly winds (Xu et al., 2009a).

### Ice Core Drilling and Sampling

On 2006, 97 and 33 m length ice cores (12 cm diameter) were drilled from ZQP and NJKS, respectively, and then transported frozen and processed in a cold room at −20°C. The samples were processed as described in Liu et al. (2016b). Half of ice core was used for microbial analyses, and the remaining half was used for physicochemical analysis. In the sterile environment, the ice cores were cut into 10–20 cm long segments and outer ring was sawed off 1 cm to decontaminate. After decontamination, samples were placed in the sterile containers and melted at 4°C (Christner et al., 2005).

### Chemical Analysis and Dating

The concentration of water-insoluble organic, elemental, and total carbon of BC samples was carried out as described in Xu et al. (2009b). Briefly, ice cores were cut lengthways into four columns. Every column was cut at intervals 10–20 cm into segments and used for chemical measurements. After melting, the liquid sample was filtered through a quartz fiber filter, ensuring the uniform particles were distributed on the surface of filter paper, then oven-dried at 40°C for 6 h. The oven-dried samples were then transferred to a glass vacuum desiccator. Organic carbon and elemental carbon were carried out based on the Interagency Monitoring of Protected Visual Environments (IMPROVE) thermal/optical reflectance protocol. Oxygen isotope ratios, BC, and organic carbon concentrations were used to date the ice cores as described by Xu et al. (2009b).

ZQP Glacier was selected to compare the seasonal variations. Annual precipitation of samples was measured at the nearest meteorological station. The annual water equivalent precipitation from 1960 to 2006 averaged 797 mm at the drill site. Glaciers in this region were reported as “spring accumulation,” and the largest precipitation happens in the April as meteorological station recorded. Consider the glacier accumulation and monsoon occurrence time, a three-season approach was used based on the annual accumulation regime, with the pre-monsoon season from January to May, monsoon season from June through September, and the post-monsoon season from October through December.

### Flow Cytometry and Measuring LNA (HNA) Functional Groups

Staining and FCM were carried out to quantify total cell concentration and abundance of LNA (HNA) functional groups based on the methods described previously (Hammes et al., 2008; Prest et al., 2013). The basic principle of FCM measurements is that bacteria are stained with fluorescent dyes in order to distinguish them from background (e.g., BC and other particles; Prest et al., 2013). Ice core melt water was fixed with 1% glutaraldehyde incubated for 10 min and analyzed within 8 h. Sample volumes of 1 ml were stained with 10 μl SYBR® Green I [1:100 dilution in .20-μm-filtered dimethyl sulfoxide (DMSO), Invitrogen]. The samples and dye were mixed by a brief vortex and then incubated for 10 min in the dark at 37°C before measurement.

Measurements were performed using an EPICS ALTRA II flow cytometer (Beckman Coulter, United States) equipped with a 100-mW water-cooled argon-ion laser, emitting at a fixed wavelength of 488 nm. Bacterial signals were triggered on green fluorescence. The multi-parameter data were analyzed as follows. First, bacterial cells were selected using fixed gating on the two-parameter dot plots of green fluorescence (FL1-H; 530 nm) versus red fluorescence (FL3-H; >670 nm). Next, two fixed gates were applied to separate LNA from HNA functional groups using the same two-parameter dot plots as described by Prest et al. (2013). Triplicate samples were measured. Total cell concentration and abundance of LNA (HNA) functional groups were averaged over the three replicates. The corresponding optical signals are converted into electronic signals. The output data were processed using the CytoWin 4.1 software.^1^

### Statistical Analysis

The correlation between HNA and LNA functional groups was tested by running ordinary least squares regression (Rubbens et al., 2019). To explore the time trends of LNA/HNA ratio, Poisson generalized additive models (GAMs) were used to model (Liu et al., 2016b). The GAMs accounts for the Poisson distribution of cell counts, the detected over-dispersion of the response, and the autocorrelation expected in time series. The GAMs allow exploration of non-linearities between the responses and explanatory variables and allow specification of the distribution of the response variable (bacterial abundance) by a function (Marra and Wood, 2011). Differences in non-monsoon and monsoon samples data were analyzed using parametric tests. Permutational multivariate analysis of variance (PERMANOVA) was used to investigate differences in abiotic and biotic variables between two season samples. Correlations were performed to test the associations LNA/HNA ratio and bacteria abundance with environmental variables and BC factors using vegan package. Procrustes test and Mantel test (Dixon, 2003) were used to explore the relationships between BC factors and proportion of LNA and HNA functional groups in different seasons with R package vegan. Monte Carlo value of *p* for rotational agreement significance testing was determined from 999 permutations. Variation partition analysis (VPA) was used to delineate the effects of environmental and BC components on variation of LNA/HNA ratio. The above-mentioned statistical analyses were performed using R (v3.4.3).

## Results and Discussion

### Dominance of LNA Functional Groups in Ice Cores

Fluorescent fingerprinting based on FCM data is a robust and standardized approach to identify LNA and HNA functional groups (Prest et al., 2013; Proctor et al., 2018; Santos et al., 2019). The reproducible staining methods and the fixed FCM gate were applied for all samples to ensure comparable results. Both the LNA and HNA functional groups were identified in Tibetan ice cores (Supplementary Figure 2), consistent with other ecosystems (Bouvier et al., 2007; Bowman et al., 2017; Proctor et al., 2018). The abundance of LNA functional groups and HNA functional groups was significantly correlated in both the NJKS and ZQP glaciers (ordinary least squares regression, adjusted *R*^2^ = .67, *p* < .001). This suggests that the bimodal distribution of fluorescence intensity could be ubiquitous in Tibetan glaciers. Furthermore, the mean proportion of LNA functional groups to total cell counts was 62.4% ± 7.2% (Supplementary Figure 3), which is a typical value for oligotrophic ecosystems (Wang et al., 2009; Kaartokallio et al., 2013). LNA functional groups could survive and withstand limited nutrient environments due to their high affinity and binding-protein dependent uptake system (Salcher et al., 2011). In addition, LNA functional groups can adopt a dormancy strategy to withstand limited nutrient concentration (La Ferla et al., 2014). LNA functional groups have small microbial cell sizes (Proctor et al., 2018). The abundant microbial population in Greenland glacier ice core was dominated by small cells (Miteva and Brenchley, 2005). Small cell size is advantageous for more efficient nutrient uptake in oligotrophic conditions due to a larger surface-to-volume ratio, protection against predators, and occupation of microenvironments (Miteva and Brenchley, 2005). Consistently, Santos et al. (2019) pointed out that LNA functional groups are biomarkers of nutrient-limited environments.

### Total Cell Concentration Was Proportional to the BC

**Figure 1:**
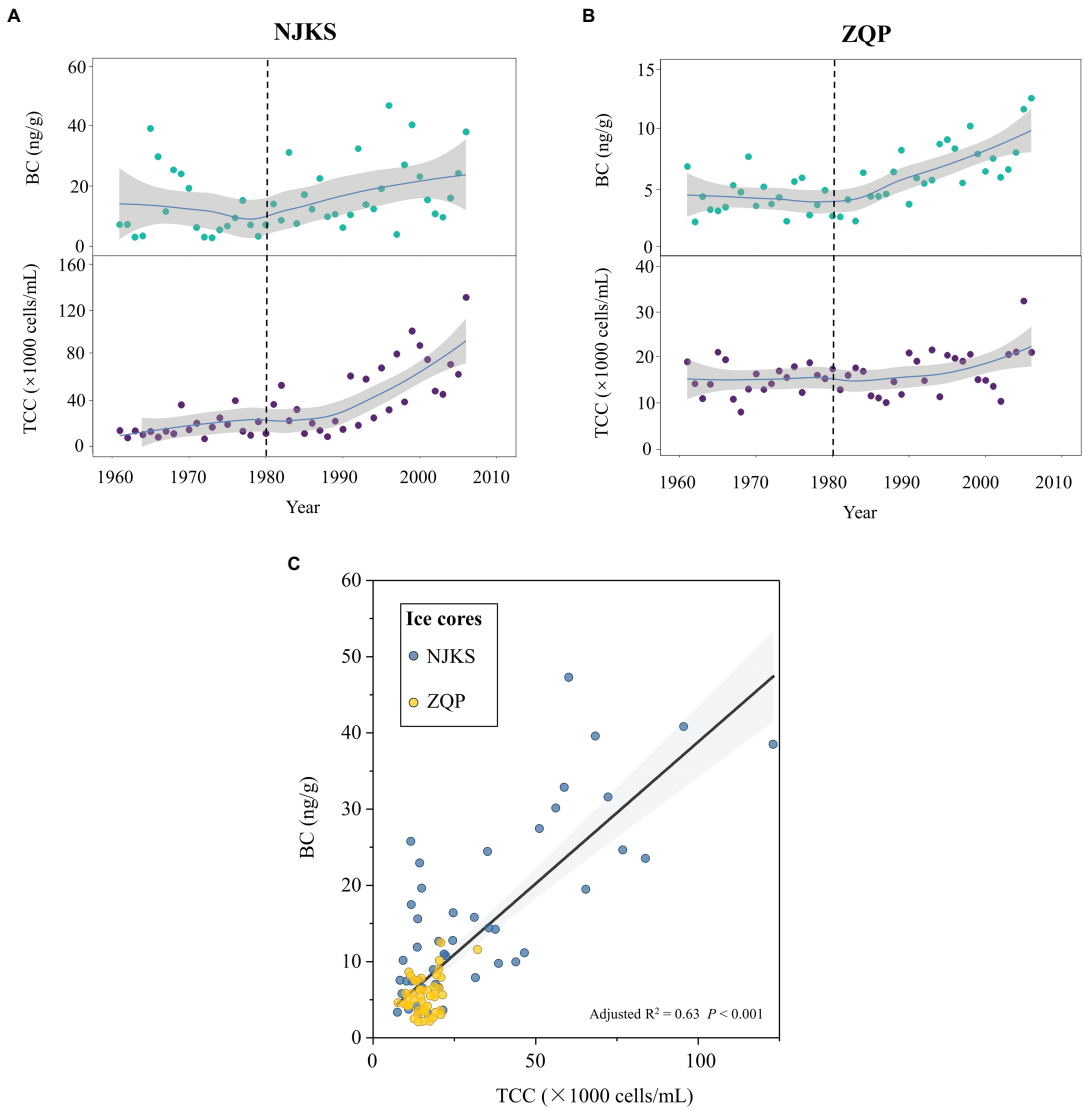
Temporal variation of total cells concentration (TCC) and black carbon (BC) concentration from 1960 to 2006 in Noijinkangsang (NJKS) and Zuoqiupu (ZQP) glacier ice cores and their correlation. Time trends (solid lines) of TCC and BC in NJKS **(A)** and ZQP **(B)**. All trends were estimated using GAMs. The gray-shaded areas in the graphs represent the 95% CIs. **(C)** Correlation between TCC and BC across the two glacier ice cores. The gray-shaded areas in the graphs represent the 95% CIs.

TCC as a proxy has often been used to assess bioavailable carbon in the environment (Elhadidy et al., 2016), and it also reflected the level of nutrients (Sharuddin et al., 2018; Qi et al., 2021). In the temporal scale, TCC and BC displayed a similar increasing trend in NJKS and ZQP, respectively. BC and TCC remained relatively stable or slightly increased in 1960–1980; however, a rapid ascending pattern was observed after 1980 until 2006 (Figures 1A,B). We also found a positive relationship between the BC and TCC in both the NJKS and ZQP glaciers (ordinary least squares regression, adjusted *R*^2^ = .63, *p* < .001; Figure 1C). There might be two reasons for the increasing of TCC: (i) bacteria were brought onto the glacier by a carrier of BC. This finding was consistent with previous studies (Zhang et al., 2007; Yao et al., 2008; Liu et al., 2016b), which demonstrated atmospheric deposition was responsible for transporting bacteria and (ii) dissolved BC boosted accumulation of nutrient concentration on the glaciers and was available for bacterial reproduction during post-depositional process (Santibanez et al., 2018). Previously BC was considered recalcitrant and inaccessible for bacteria; however, BC is very reactive and oligotrophic bacteria can consume as a carbon source for their growth (Hartnett and Hamilton, 2016). It has been reported that UV light could stimulate chemical changes in BC and making it easier for degradation by microorganisms (Malits et al., 2015). Since most glacial regions receive maximum light all year round (Zheng et al., 2000; Shen et al., 2015), the levels of BC degradation will thereby increase resulting in increment of bacteria abundance.

### Temporal and Seasonal Variation of LNA (HNA) Functional Groups

#### Temporal Variation

**Figure 2:**
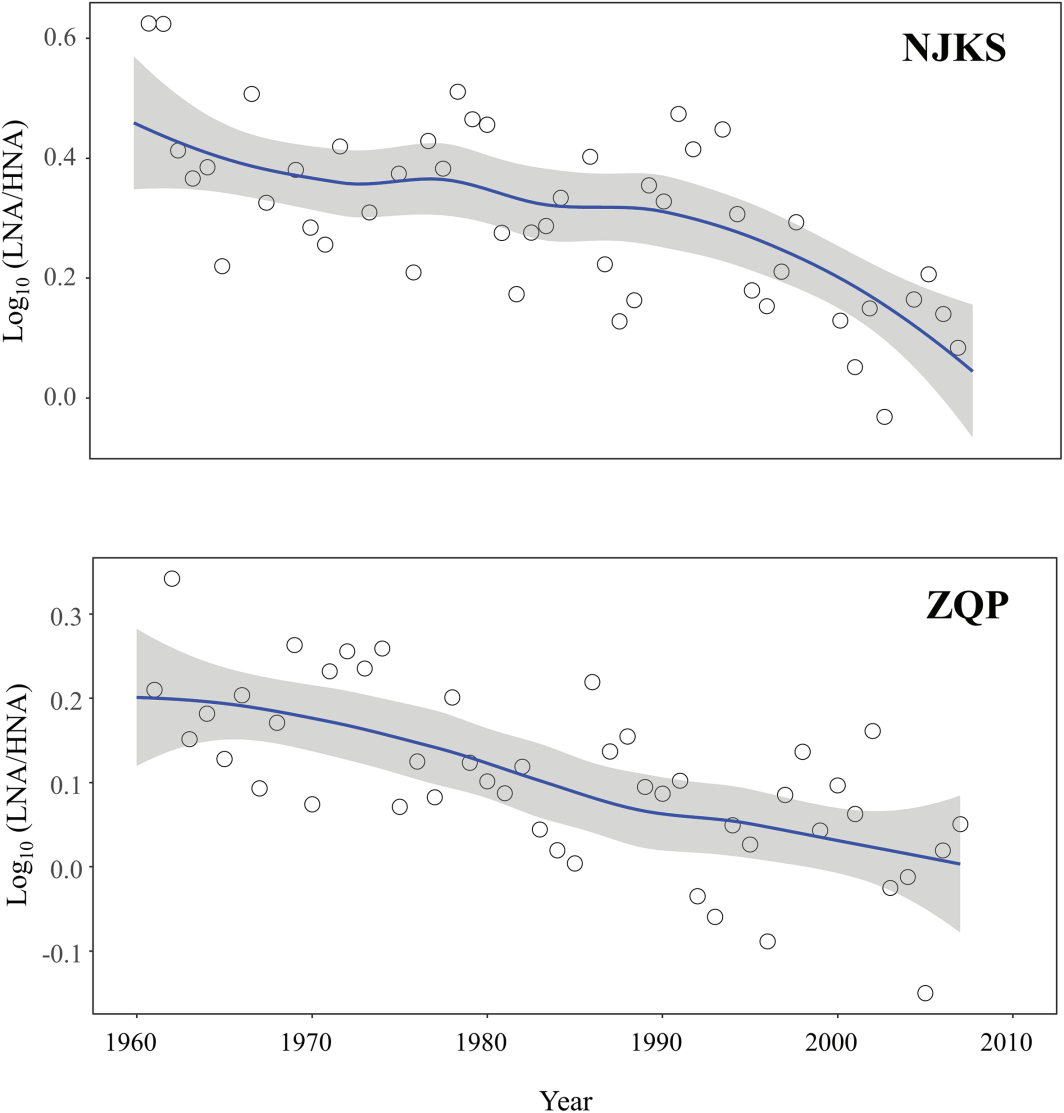
Time trends (solid lines) of Log_10_ (LNA/HNA) in NJKS (edf = 8.19) and ZQP (edf = 5.49) glacier ice cores. All trends were estimated using GAMs. The gray-shaded areas in the graphs represent the 95% CIs.

**Figure 3:**
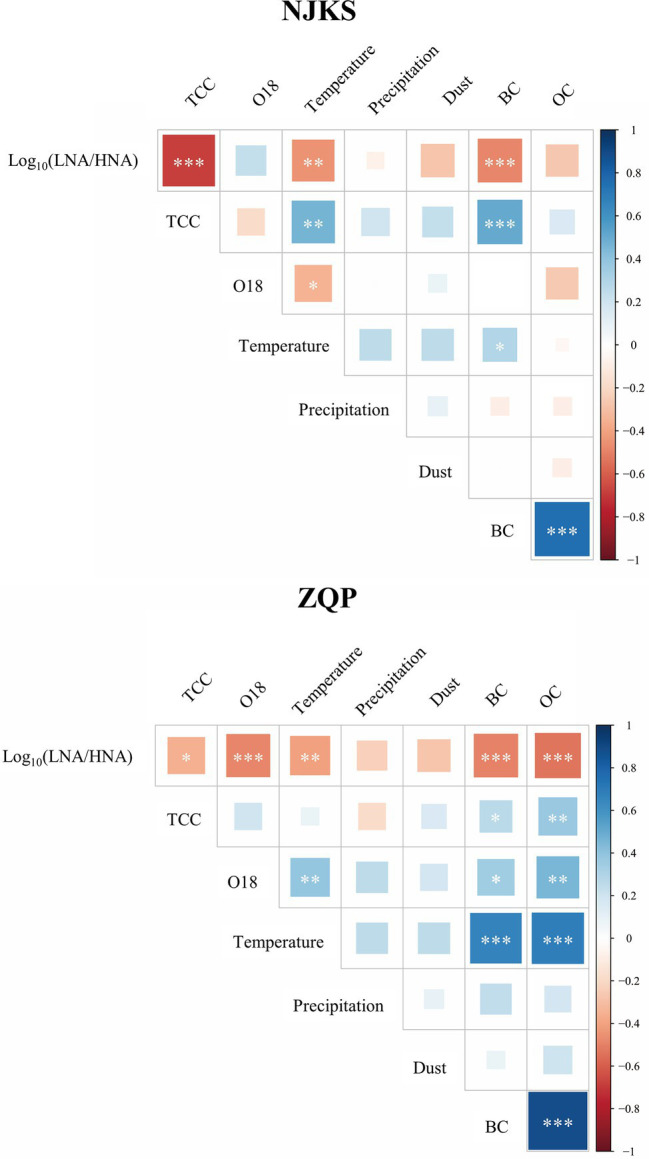
Heatmaps showed the correlation of abiotic and biotic variables in NJKS and ZQP. Correlation coefficient denoted as *p* < .05 (^*^), *p* < .01 (^**^), and *p* < .001 (^***^).

The abundance ratio of LNA-to-HNA functional groups decreased for the period examined (Figure 2), and the generalized additive model (GAM) analysis showed that the reduction was statistically significant (*p* < .05). Typically, the ratio of LNA functional groups was higher than HNA functional groups in oligotrophic conditions with minimum contamination (Servais et al., 2003; Salcher et al., 2011). On the other hand, compared with LNA functional groups, HNA functional groups are more sensitive to changes in nutritional environment (Santos et al., 2019). In other words, HNA functional groups highly related to increment of nutrients (Sharuddin et al., 2018). It should be noted that BC (*p* < .001) significantly and negatively correlated with the LNA-to-HNA ratio in both glaciers (Figure 3), which suggested that a dissolved fraction of BC could be responsible for the reduced LNA-to-HNA ratio. It is worth noting that a given bacterium can be sometimes categorized as LNA functional groups and sometimes HNA functional groups depending on environmental conditions (Wang et al., 2009; Martinez-Garcia et al., 2012). This classification inconsistency is mainly since LNA functional groups should be dormant under oligotrophic conditions, but then switch to an active condition following organic loading (Sharuddin et al., 2018; Santos et al., 2019). In fact, this feature can better explain the bioindicator value of LNA-to-HNA ratio since they sensitively respond to fluctuations in environmental conditions rather than they are only dependent on the community taxon composition.

#### Seasonal Variation

**Figure 4:**
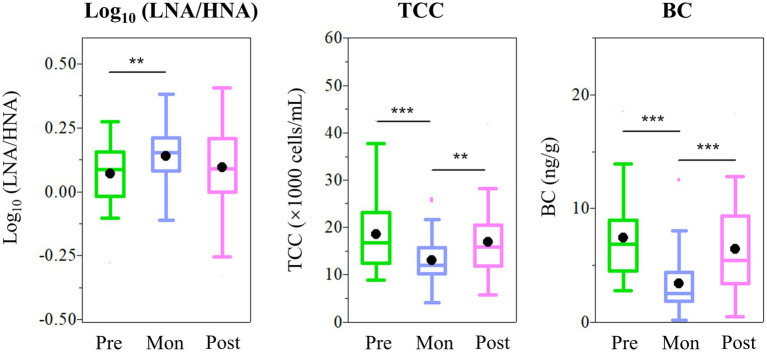
Seasonal variation of Log_10_ (LNA/HNA), TCC and BC concentration in ZQP. Correlation coefficient denoted as *p* < .05 (^*^), *p* < .01 (^**^), and *p* < .001 (^*^**).

Seasonal fluctuation in the LNA-to-HNA ratio was also observed in the ZQP Glacier. O18 isotopic ratio, an Indian monsoon precipitation proxies (Yu et al., 2021), exhibited a negative correlation with the LNA-to-HNA ratio significantly (*p* < .001, Figure 3). As shown in Figure 4, LNA functional groups were more prevalent in the monsoon samples with an average LNA-to-HNA ratio of .14 ± .11 (Figure 4). Bacteria deposited during the monsoon period are partly originated from the Indian Ocean, which is dominated by LNA functional groups numerically (Zubkov et al., 2006). This could explain the higher LNA ratio during the monsoon season. The TCC also showed seasonal variation. TCC in monsoon samples (12.9 ± 4.7 × 10^3^ cells/ml) was significantly lower (*p* < .01 or *p* < .001) than those in the pre-monsoon (18.4 ± 7.5 × 10^3^ cells/ml) and post-monsoon samples (16.5 ± 9.3 × 10^3^ cells/ml), while no significant difference was found between pre-monsoon and post-monsoon samples. This seasonal variation in the distribution of bacteria is related to the atmospheric circulation on the Tibetan Plateau (Yao et al., 2008). Specifically, a higher TCC was observed during the non-monsoon season, consistent with the higher atmospheric deposition loading brought by the westerlies (Kang et al., 2000).

Increasing BC on the Tibetan Plateau could influence bacterial distribution. BC variation exhibited an opposite trend of a lower concentration during the monsoon season (3.41 ± 2.47 ng/g) and higher during the pre-monsoon (7.38 ± 3.46 ng/g) and post-monsoon periods (6.39 ± 3.87 ng/g; Figure 4). There was no significant different between pre-monsoon and post-monsoon. Environmental factors can also affect the LNA-to-HNA ratio. Temperature exhibited a negative correlation with the LNA-to-HNA ratio significantly (*p* < .01; Figure 3). The temperature dependence of metabolic rates of bacteria in deep glacier ice was for survival of imprisoned bacteria (Price and Sowers, 2004). High temperature leaded to more activity bacteria, as recorded in the ice core. With principal coordinate analysis (PCoA), the pre-monsoon and post-monsoon samples were clearly clustered into one group and separated from the monsoon samples (Supplementary Figure 4), which was confirmed by dissimilarity test (PERMANOVA, *p* < .001).

### Correlation Between BC and LNA and HNA Functional Groups

**Figure 5:**
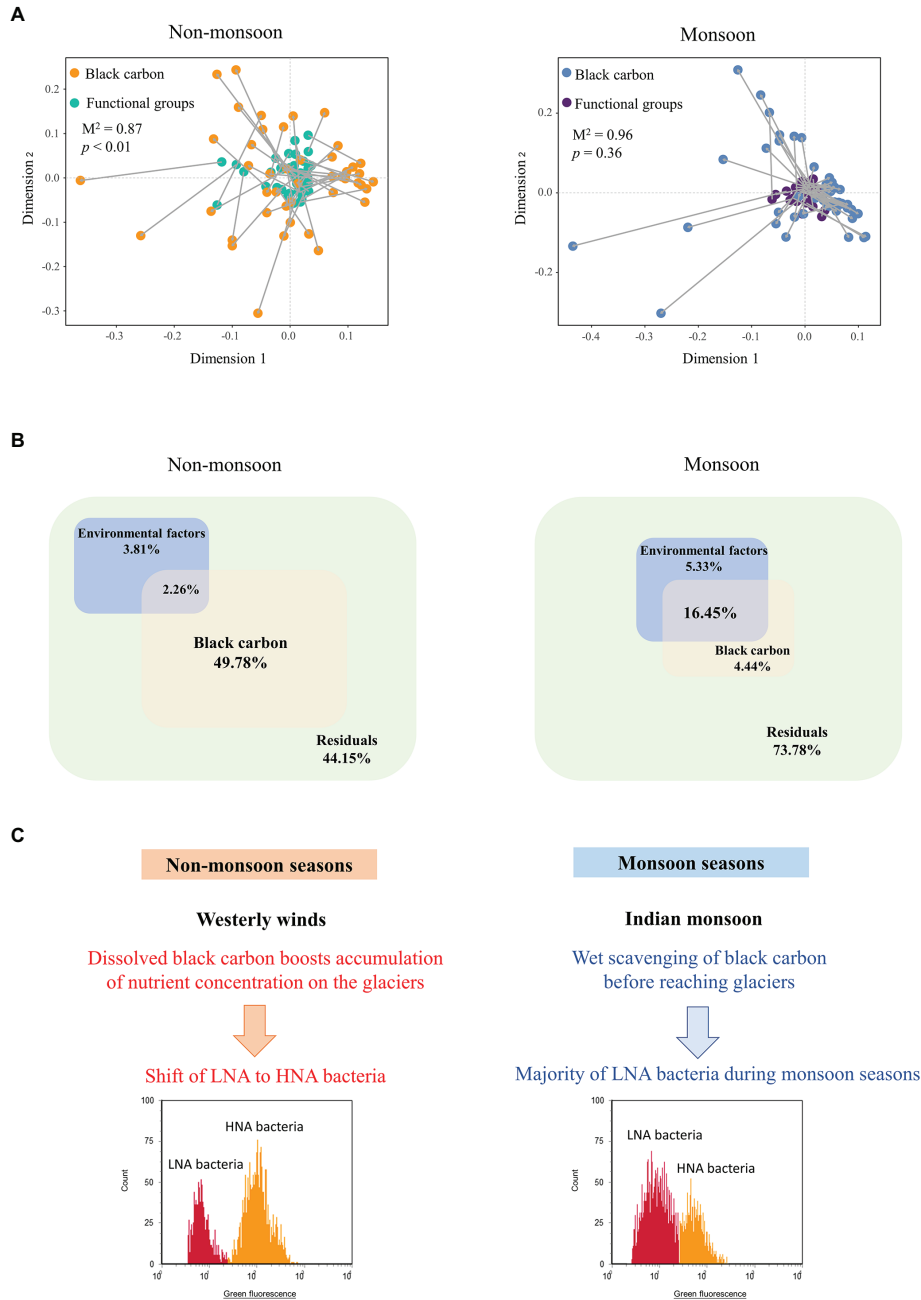
Relationships between BC and LNA and HNA functional groups. **(A)** Procrustes analyses of the correlations between BC and functional groups during non-monsoon and monsoon. *M*^2^ values represent the Procrustes sum of squares. Value of *p* represents the significance under 999 permutations test. **(B)** Variation partitioning analysis (VPA) differentiating the effect (%) of environmental factors and BC on proportion of LNA and HNA. **(C)** Schematic representation of HNA and LNA functional groups respond to BC under influence of Indian Monsoon.

We applied Procrustes analysis to test for functional groups and BC factors across seasonal samples. Our analysis showed that BC variations correlated with functional groups using Euclidean distances in the non-monsoon season (Figure 5A, Procrustes, Monte Carlo *p* < .01, 999 permutations). This was consistent with the Mantel test results (*r* = .22, *p* = .003). Interestingly, we did not find a similar correspondence between BC variations and functional groups during the monsoon period (Figure 5A, Procrustes, Monte Carlo *p* = .36, 999 permutations). Variation partitioning analysis (VPA) further differentiated the contributions of BC factors and environmental factors on functional groups’ variations (Figure 5B). The BC (49.78%) showed a greater contribution to functional groups’ variations in non-monsoon. In comparison, BC only explained 4.44% for the functional groups’ variations in the monsoon season. The joint effects of multiple factors explained 2.26% and 16.45%, leaving 44.15% and 73.78% of functional groups’ variations in the non-monsoon and monsoon seasons, respectively. Both the Procrustes analysis and VPA results demonstrated that potential causal relationships may occur between LNA (HNA) functional groups and BC factors in the non-monsoon season.

A scheme illustrating how two functional groups respond to BC under influence of westerly winds and Indian Monsoon was proposed to explain the pattern observed (Figure 5C). During non-monsoon seasons, BC deposited onto the southeastern Tibetan Plateau by the westerly winds (Xu et al., 2009a). UV light could stimulate chemical changes in BC, leaching of dissolved BC boosts accumulation of nutrient concentration on the glaciers (Malits et al., 2015). The diverse characteristics of BC and high reactivity have the potential to alter microbial biomass growth, community structure, and activity in many ways. Bacteria transition from death or a dormant state (primarily LNA functional groups) to active growth (primarily HNA functional groups) in the nutrient-rich condition (Bouvier et al., 2007; Wang et al., 2009). Previous results have indicated that an immediate increase in bacterial abundance and metabolically activity in glaciers after BC deposition (Liu et al., 2016b; Santibanez et al., 2018). BC could adsorb organic matter and nutrients and serve as a nutrient reservoir for microorganisms (Busscher et al., 2008; Weinbauer et al., 2012).

In comparison, BC was lower during the monsoon seasons (Figure 4). This could be due to the high atmospheric moisture of the Indian monsoon that enhanced precipitation of BC before it reached glaciers (Xu et al., 2009a; Yang et al., 2021). LNA functional groups are dominant in nutrient-limited environments on account of their high nutrient acquisition efficiency (Sharuddin et al., 2018; Santos et al., 2019). Thereby, organic loads are crucial factors influencing the LNA-to-HNA ratio. This has been proposed previously that trophic statuses of the growth conditions were associated with LNA/HNA ratio (Santos et al., 2019). In this study, the higher proportion of HNA functional groups during the non-monsoon season could be related to their eutrophic status (Servais et al., 2003). This finding aligns with the microbial biogeography study of the same glacier that Actinobacteria, which is categorized as “overlapped” HNA (i.e., sometimes categorized as HNA and sometimes LNA; Vila-Costa et al., 2012; Proctor et al., 2018), was the dominant phylum with increasing BC concentrations (Liu et al., 2016b). The “overlapped” functional groups depend on environmental condition. The hypotheses of Bouvier et al. (2007) could explain “overlapped” functional groups: (i) that LNA functional groups consist of dormant groups that move to HNA when they become active in the optimal condition and (ii) that HNA functional groups consist of active groups that move into the LNA fraction after inactivation.

## Conclusion

In this study, high-resolution temporal variations of bacterial abundance and functional groups in ice cores from the Tibetan Plateau were investigated. A variation of the LNA/HNA ratio was detected, which is a measure of the transition of bacterial community from a dormancy to an actively growth. Our data identified an accumulation of nutrient concentration on the glaciers over the last half-century and demonstrated the rapid deterioration of trophic status on the sensitive ecosystem due to increment of BC. The responses of bacteria endpoints to nutritional environment caused by anthropogenic activity indicated that endless BC emissions could result in serious and irreversible impacts on trophic statuses of the Tibetan Plateau. The fluorescent fingerprinting had considerable potential application for bacterial monitoring and early-warning detection of microbiological effects of BC.

## Data Availability Statement

The original contributions presented in the study are included in the article/Supplementary Material, further inquiries can be directed to the corresponding author.

## Author Contributions

GM and YL contributed to the study conception and design. Material preparation, data collection, and analysis were performed by GM, MJ, BX, YL, and NJ. The first draft of the manuscript was written by GM and MJ. GM, MJ, BX, YL, and NJ commented on previous versions of the manuscript. All authors read and approved the final manuscript.

## Funding

This work was supported by the National Research and Development Program of China (grant no. 2019YFC1509103), the National Natural Science Foundation of China (grant nos. 91851207, 42101128, and 41988101), and the China Postdoctoral Science Foundation (2021M693254).

## Conflict of Interest

The authors declare that the research was conducted in the absence of any commercial or financial relationships that could be construed as a potential conflict of interest.

## Publisher’s Note

All claims expressed in this article are solely those of the authors and do not necessarily represent those of their affiliated organizations, or those of the publisher, the editors and the reviewers. Any product that may be evaluated in this article, or claim that may be made by its manufacturer, is not guaranteed or endorsed by the publisher.

## Footnotes

^1^ http://www.sb-roscoff.fr/phyto/cyto.html
