# Supplementary material for: Variation of High and Low Nucleic Acid-Content Bacteria in Tibetan Ice Cores and Their Relationship to Black Carbon
Source: Front Microbiol. 2022 Feb 14;13:844432. doi: 10.3389/fmicb.2022.844432 (PMC8882866; doi:10.3389/fmicb.2022.844432)
Supplement: Supplementary file 1 [file Data_Sheet_1.pdf]

Supplementary Material

Variation of High and Low Nucleic Acid-Content Bacteria in Tibetan Ice Cores and Their Relationship to Black Carbon

Guannan Mao[1], Mukan Ji[2], Baiqing Xu[1, 3,4], Yongqin Liu [1,2,3,4 *], Nianzhi Jiao[5]

[1] Key Laboratory of Tibetan Environment Changes and Land Surface Processes, Institute of Tibetan Plateau Research, Chinese Academy of Sciences, Beijing 100101, China

[2] Center for the Pan-third Pole Environment, Lanzhou University, Lanzhou 730000, China

[3] CAS Center for Excellence in Tibetan Plateau Earth Sciences, Chinese Academy of Sciences, Beijing 100101, China

[4] College of Resources and Environment, University of Chinese Academy of Sciences, Beijing 100049, China

[5] State Key Laboratory of Marine Environmental Science, Xiamen University, Xiamen 361005, China

*** Correspondence:**

Yongqin Liu; (Email) yqliu@itpcas.ac.cn

The supplementary information includes:

- **5 pages**
- **4 supplementary figures (pages 2-5)**

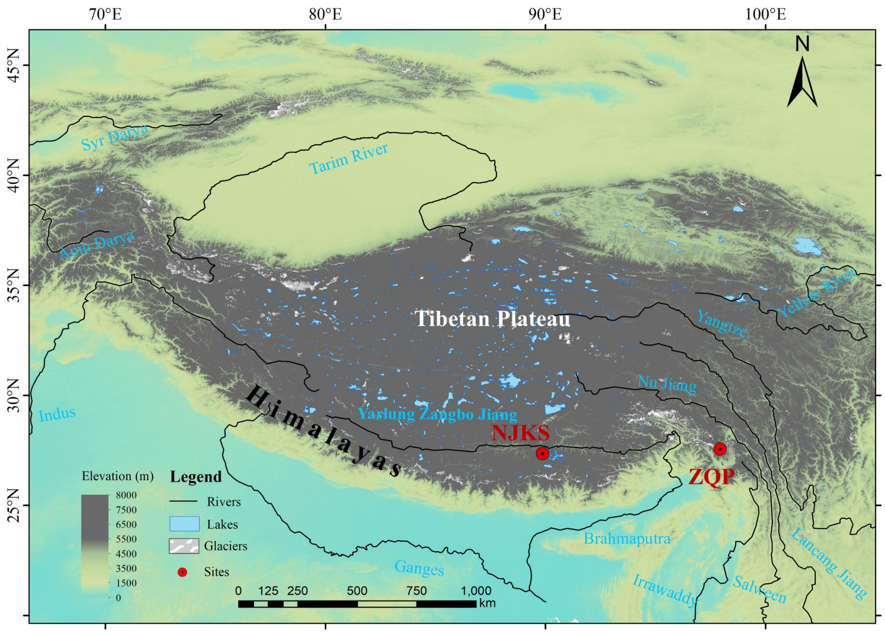

Supplementary Figure 1 Locator map of the two ice cores. NJKS is from the Noijinkangsang Glacier (90.20°E, 29.04°N, 5950 m.a.s.l.); and ZQP is from the Zuoqiupu Glacier (96.92°E,29.21°N, 5600 m.a.s.l.).

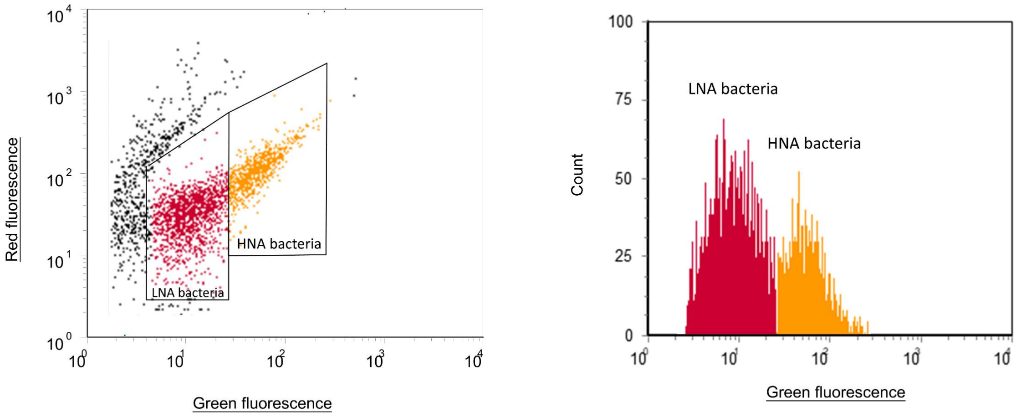

Supplementary Figure 2 Representative flow cytometry dot plots and green fluorescence histogram showing distributions of LNA and HNA functional groups in ice cores.

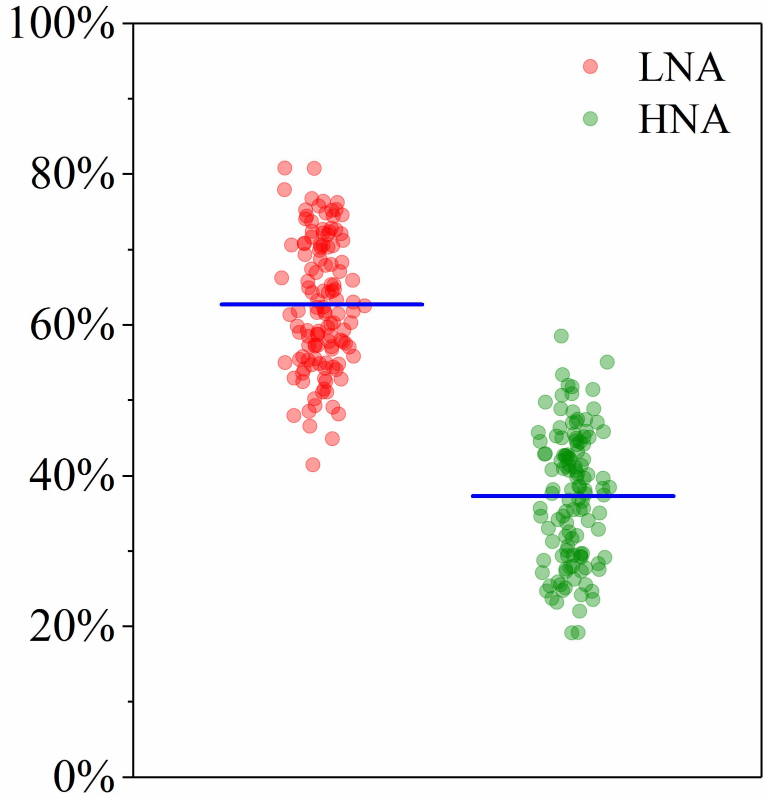

Supplementary Figure 3 Proportion of LNA and HNA functional groups in ice cores. Blue lines indicate average value.

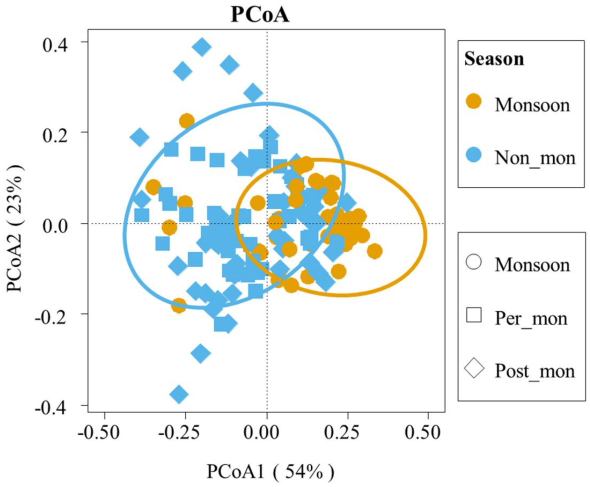

P value of difference between sample groups (PERMANOVA)

	Per_mon	Monsoon
Monsoon	**0.001**	
Post_mon	0.692	**0.001**

Supplementary Figure 4 Principal coordinate analysis (PCoA) and Permanova test of abiotic and biotic variables in ZQP among the three seasons.
